# The PERK inhibitor GSK2606414 evokes developmental defects in zebrafish consistent with Wolcott-Rallison syndrome phenotypes

**DOI:** 10.1007/s43440-026-00837-7

**Published:** 2026-02-13

**Authors:** Liliana M. Almeida, Leonor Pereira Lima, Nuno A. S. Oliveira, Rui F. O. Silva, Bruno Sousa, José Bessa, Brígida R. Pinho, Jorge M. A. Oliveira

**Affiliations:** 1 Applied Molecular Biosciences Unit, Mitochondria and Neurobiology Lab, Faculdade de Farmácia, UCIBIO, Universidade do Porto, Porto, 4050-313 Portugal; 2 i4HB, Institute for Health and Bioeconomy, Universidade do Porto, Porto, 4050-313 Portugal; 3 Instituto de Biologia Molecular e Celular, IBMC, Universidade do Porto, Porto, 4200-135 Portugal; 4 i3S Instituto de Investigação e Inovação em Saúde, Universidade do Porto, Porto, 4200-135 Portugal; 5 Departamento de Ciências do Medicamento, Laboratório de Farmacologia, Faculdade de Farmácia, Universidade do Porto, Rua de Jorge Viterbo Ferreira, 228, Porto, 4050-313 Portugal

**Keywords:** Developmental diseases, Metabolic diseases, Model organism, Zebrafish, PERK, Wolcott-Rallison syndrome

## Abstract

**Background:**

Protein kinase RNA-like endoplasmic reticulum kinase (PERK) is an endoplasmic reticulum stress kinase whose loss of function disturbs human development, leading to skeletal dysplasia and permanent neonatal diabetes, as in the Wolcott-Rallison Syndrome (WRS). The lack of effective, less invasive therapies for developmental diseases highlights the need for animal models that replicate complex pathological phenotypes, while allowing scalable drug screening. Zebrafish high fecundity and rapid development enable efficient in vivo drug testing. We assessed zebrafish’s potential for studying PERK and its pharmacological modulation in developmental diseases like WRS.

**Methods:**

To assess the similarity between human and zebrafish PERK we used bioinformatic analyses. To inhibit PERK we used GSK2606414. To evaluate effects on skeletal, neuromuscular, and cardiac development we combined behavioural and functional assays. To assess diabetic-like phenotypes we used fluorescent pancreatic markers and a glucose probe.

**Results:**

Zebrafish PERK conserves 11 of 12 critical GSK2606414‑binding residues (predicted 3D structures highly similar). Functionally, GSK2606414 (10 µM) decreased levels of PERK pathway markers and induced WRS-relevant phenotypes: reduced body length, increased trunk–tail curvature, decreased cranial cartilage staining; neuromuscular impairment (altered reflexes, reduced muscle birefringence) and cardiac dysfunction (pericardial oedema, reduced stroke volume and cardiac output). However, parameters not associated with WRS like otolith area and eye/body ratio remained unaffected. Moreover, GSK2606414 decreased 𝛽-cell mass and lowered 2-NBDG-glucose uptake in neuromasts, consistent with diabetic-like phenotypes.

**Conclusion:**

These findings evidence zebrafish’s potential for studying PERK function and its pharmacological modulation in developmental disorders like WRS, aiding research on pathophysiology and experimental treatments.

**Supplementary Information:**

The online version contains supplementary material available at 10.1007/s43440-026-00837-7.

## Introduction

Protein kinase RNA-like endoplasmic reticulum kinase (PERK) is an endoplasmic reticulum (ER) membrane kinase that activates in response to disruptions in ER protein homeostasis (proteostasis) by promoting a protective response, the integrated stress response (ISR). Upon activation by stress, PERK phosphorylates eukaryotic translation initiation factor 2α (eIF2α), which decreases global protein synthesis, alleviating nascent protein load [1] and boosts the synthesis of transcription factors like activating transcription factor 4 (ATF4) and C/EBP homologous protein (CHOP). These transcription factors regulate the expression of chaperones, proteases, and components of the autophagic pathway, thus contributing to proteostasis maintenance [1]. During development, protein synthesis plays a critical role in cell division, differentiation, and the rapid growth of embryos [2], and PERK is essential for detecting protein misfolding and adjusting protein synthesis to maintain proteostasis [3].

In humans, loss-of-function mutations in the *EIF2AK3* gene encoding for PERK cause a rare (prevalence of < 1/1 000 000; ORPHA:1667) autosomal recessive developmental disease named Wolcott-Rallison Syndrome (WRS), with patients dying before 35 years of age [4]. WRS is mainly characterized by skeletal dysplasia and neonatal diabetes mellitus [5], but WRS patients may also present broad symptomatology like motor neuropathy, intellectual deficit, early neurodegeneration, and cardiac malformations [4]. This broad symptomatology is likely due to the widespread distribution of PERK, with several loss-of-function mutations totally or partially abolishing its kinase activity [4, 6]. Existing therapies for WRS patients include blood glucose control with insulin pumping and transplant of liver/pancreas and kidney [7]. The scarcity of non-invasive and more effective therapeutic options for WRS patients highlights the need for further therapeutic investigation, which requires efficient WRS models.

The currently available in vivo models for the developmental disease WRS are rodents with PERK knock-out or loss-of-function mutations in PERK that exhibit the WRS hallmarks – neonatal diabetes and skeletal dysplasia [8, 9] – and the broad-spectrum phenotypes – cardiac, muscle, and cognitive abnormalities [10–12]. However, for large exploratory studies of new therapeutics, there are efficiency advantages in using small vertebrates like zebrafish before testing the most promising candidates in rodent models. Zebrafish are increasingly used to develop disease models given their advantageous features: high fecundity, external fertilization, and rapid formation of complete organs, making zebrafish ideal for investigating developmental diseases and for rapid and scalable in vivo drug testing [13, 14]. Previous studies that targeted PERK in zebrafish used either genetic (morpholino knockdown) or pharmacological inhibition [15–17]. In such studies, PERK inhibition with GSK2606414 was used in zebrafish adults to study sleep regulation [15], and PERK inhibition with GSK2656157 or PERK knockdown was used to study redox regulation and neurogenesis in zebrafish embryos [16, 17]. To the best of our knowledge, there are no previous studies in zebrafish with a primary focus on studying PERK function in developmental diseases, including WRS.

Here, we combined bioinformatic analyses, molecular biology techniques, behaviour, and functional assays to assess the effects of pharmacological PERK inhibition in zebrafish. We used transgenic zebrafish expressing pancreatic reporters and a fluorescent glucose probe to assess diabetic-like phenotypes. We performed functional and structural microscopy to assess skeletal, neuromuscular, and cardiac development. Our data show that zebrafish holds potential as a model to study PERK function in developmental disorders, such as WRS.

## Materials and methods

### Drugs, solvents, and solutions

Stock solutions of the PERK inhibitor GSK2606414 (Merck Millipore, 516535) [15, 18], the muscle relaxant d-tubocurarine (dTC; Sigma, T2379), and the anesthetic tricaine methanesulfonate (0.02%; Sigma, E10521) were prepared in DMSO (Sigma, 276855) and diluted in standard E3 medium [19]. DMSO levels were kept under 0.2% [20].

### Zebrafish maintenance and husbandry

Adult AB strain zebrafish were supplied by the Interdisciplinary Centre of Marine and Environmental Research (CIIMAR, Portugal), and transgenic strains of zebrafish were obtained from José Bessa’s laboratory at the Institute for Research and Innovation in Health (i3s, University of Porto, Portugal). Zebrafish adults were maintained at 28 ± 1 °C on a 14 h:10 h light: dark cycle [21, 22]. Breeding pairs (1:1, male: female) were placed in breeding tanks on the day before egg collection. Ninety minutes after starting the light period, eggs were collected, and the time point was recorded as 0 h post fertilization (hpf). Ethics statement: Handling of zebrafish followed the European Directive 2010/63/EU and the Portuguese Law (Decreto-Lei 113/2013). Experiments were performed according to the 3Rs principle by reducing the number of animals used and using refined techniques that reduce animal suffering, using procedures approved by the i3S Animal Welfare Body (document ID: 2022-36, date of Issue: 2023-06-13, institution: Organismo Responsável pelo Bem Estar Animal (ORBEA) do Instituto de Investigação e Inovação em Saúde (i3S) da Universidade do Porto, Portugal).

### Drug treatments

5 zebrafish/well were randomly distributed in 24-well plates (0.5 mL E3/well) and kept at 28 ± 1 °C on a 14 h:10 h light: dark cycle throughout the assay. Zebrafish were continuously exposed to GSK2606414 or solvent (DMSO) from 4 to 76 hpf, and solutions were renewed daily [21]. dTC was incubated for 10 min before assays endpoint (≥ 15 zebrafish).

### Brightfield microscopy

For brightfield microscopy, we used a stereomicroscope (Stemi DV4, Zeiss, Göttingen, Germany) or an inverted microscope (Eclipse TE300, Nikon, Tokyo, Japan). The inverted microscope was attached to a motorized stage (ProScan, Prior) and a CCD camera (ORCA-ER, Hamamatsu), constituting a Nikon-Prior-Hamamatsu (NPH) imaging system that was controlled by the open-source software Micro-Manager (v. 2.0; https://micro-manager.org):

#### Monitoring zebrafish development

Zebrafish were scored as normal, abnormal, or dead; hatched (ruptured chorion and free tail movement) or unhatched at 28, 52, and 76 hpf. Death was ascertained according to OECD guideline 236 [23], normal development as previously described [24], and abnormalities, such as cardiac and skeletal (further into scoliosis, curly-up tail, or kyphosis). Dead zebrafish were removed at each time point, and only live ones were used in subsequent assays.

#### Morphometric analysis

Zebrafish body length was measured from the tip of the head (between the eyes) to the end of the tail [25], and trunk-tail angle was measured at the intersection between the rostral (trunk) and caudal (tail) notochord at the level of the anus.

#### Heartbeat and atrio-ventricular coordination

Heartbeats were counted in 4 zebrafish per condition, per experiment, during 20 s, at 72–80 hpf [26]. Atrio-ventricular coordination recordings were obtained with “Plot Z-axis profile” on ImageJ after imaging zebrafish for 90 s at 12 frames per second.

#### Tail coilings

Spontaneous activity was measured at 28 hpf in glass-bottom 96-well plates where each zebrafish was imaged for 3 min at a frequency of 4.5 frames per second and 2 × 2 or 4 × 4 binning [19].

#### Birefringence analysis

76 hpf zebrafish were anesthetized, immobilized on a glass surface, oriented laterally, and illuminated with linearly polarized light (Screen-Tech, ST-38). An orthogonal polarization filter was placed after the samples to block directly transmitted light. Thus, only light whose polarization angle is affected by the sample is collected by the NPH imaging system [27]. Pixel intensity in the trunk region was measured by selecting the region of interest with the ImageJ software.

#### Alcian blue staining

We adapted the cartilage staining protocol from [28, 29]. Briefly, 76 hpf zebrafish were euthanised with tricaine and fixed overnight in 4% paraformaldehyde at 4 °C. Fixed zebrafish were washed with PBS containing 0.1% Tween-20 and dehydrated with 25%, 50%, 75%, and 95% ethanol in PBS. Following dehydration, zebrafish were stained overnight at room temperature with 0.1% alcian blue (Sigma-Aldrich, 101647) prepared in 70% ethanol and 1% (0.27 M) hydrochloric acid (HCl). Stained zebrafish were washed with 100% ethanol for 20 min, then rehydrated with an inverse ethanol series (95%, 75%, 50%, and 25%) containing 1% HCl. Zebrafish were then washed with PBS, digested with 0.05% trypsin-EDTA, bleached with 1% hydrogen peroxide/0.5% potassium hydroxide, and mounted in 50% glycerol for brightfield imaging with the NPH imaging system.

### Touch-evoked escape response

At 76 hpf, at least 6 zebrafish/condition were submitted to 10 alternate touches to the head and to the tail with a 10 µL pipette tip with a recovery time of 30 s [30]. Data are presented as response rates in percentage of total touches.

### Fluorescent microscopy

Zebrafish were imaged with the NPH system coupled to a monochromator (Polychrome II, Photonics) as follows.

#### 2-NBDG labelling

3 h before 76 hpf end-point, zebrafish were incubated with a fluorescent glucose analogue − 600 µM 2-NBDglucose (2-(7-Nitro-2,1,3-benzoxadiazol-4-yl)-D-glucosamine) (Tebu-Bio,10-1459) [31, 32]; and a positive control for neuromast labelling - Mitotracker Deep Red (1 µM, Invitrogen, M22426, labels polarized mitochondria in neuromasts) in 96-well plates (4 zebrafish /well). At 76 hpf, each zebrafish was anesthetized and imaged in a glass-bottom chamber. 2-NBDglucose was excited at 475 nm and Mitotracker Deep Red at 650 nm for 1 s. Emissions were collected using band pass filters (Chroma 69008 – ET – ECFP/EYFP/mCherry) with a 10 × objective. Images of the entire fish were stitched with the GNU image manipulation program (The GIMP Development Team, 2019; https://www.gimp.org) before evaluation of anterior and posterior neuromasts [33].

#### Analysis of pancreatic development

Double transgenic zebrafish expressing GFP in insulin-producing β cells and mCherry in somatostatin-producing δ cells (Tg(*ins*: *GFP*, *sst*:*mCherry*)) or in elastase-producing cells (Tg(*ins*: *GFP*, *ela*:*mCherry*)) were kept in 24-well plates (5 zebrafish/plate) with E3 media. We used two pharmacological approaches: (1) GSK2606414 exposure from 4 hpf (before the principal islet of Langerhans is formed within the endocrine pancreas) to 28, 76, or 148 hpf; (2) GSK2606414 exposure from 76 hpf (when the principal islet is already formed) to 148 hpf [34]. At the final time point (28, 76, or 148 hpf), zebrafish were anesthetized by rapid cooling on ice and fixed in 4% paraformaldehyde overnight. GFP was excited at 488 nm, and mCherry was excited at 575 nm. Emissions were collected using band pass filters (Chroma 59022 – EGFP/mCherry) with a 20 × objective. The area of GFP- or mCherry-labelled pancreas in each image was assessed with the ImageJ software (https://imagej.nih.gov/ij).

#### Cardiac function analysis

Cardiac function parameters, namely heart rate, stroke volume, and cardiac output, were studied by imaging transgenic zebrafish that express green fluorescent protein in the cardiac muscle, Tg(*hsp70l*: *DsRed2*, *cmlc2*:*EGFP*). The beating hearts of 76 hpf zebrafish were captured for 20 s at 15 frames per second with the NPH imaging system. The number of heartbeats in each video was counted and divided by the video length in minutes, to calculate the heart rate in beats per minute. To calculate stroke volume, we used a previously described approach [35]. Briefly, we approximated the cross-section of the heart ventricle, imaged along the sagittal plane, to an ellipse, and measured its dimensions (major and minor axes) using ImageJ [36]. Assuming the ventricle to be a prolate spheroid, its volume can be calculated as $$\begin{aligned}&\:\frac{4}{3}\pi\:\times\:\left(\frac{major\:axis}{2}\right){\times\:\left(\frac{minor\:axis}{2}\right)}^{2}\cr&\approx\:0.5\:\times\:\left(major\:axis\right)\times\:{\left(minor\:axis\right)}^{2}\end{aligned}$$. Stroke volume was calculated as the difference between the diastolic and systolic ventricular volumes, and cardiac output as the product of heart rate and stroke volume.

### Western blot

24–27 zebrafish larvae were rinsed with ice-cold water twice before being placed in RIPA buffer (50 mM Tris (NZYTech, MB01601), 150 mM NaCl (Merck, S9888), 1 mM EDTA (Sigma-Aldrich, E6758), 1% IGEPAL CA-630 (Sigma-Aldrich, I3021), 1% sodium deoxycholate (Sigma-Aldrich, D6750), 0.1% sodium dodecyl sulfate (Sigma-Aldrich, L3771) supplemented with a protease and phosphatase inhibitor cocktail (Thermo Scientific, 78440). After 3 freeze-thaw cycles, homogenization with Precellys Evolution (6800 rpm, 5 × 42 s with 37 s interval) and centrifugation at 12,000 × g (10 min, 4 °C), supernatant protein concentrations were quantified by the Bradford method. 30 to 40 µg of protein extracts were supplemented with Bolt LDS sample buffer (Invitrogen, B0007) and Bolt sample reducing agent (Invitrogen, B0009), denatured at 95 °C for 5 min, and loaded into 4–15% Mini-PROTEAN TGX™ Precast Protein Gels (Biorad, 4561084), which were electrophoresed (125 and 200 V, 30 min) and wet-transferred to PVDF membranes (Millipore, IPVH00010) overnight (30 V, 4 °C). Membranes were blocked (1 h, room temperature or overnight at 4 °C) with 5% bovine serum albumin (BSA, NZYTech, MB04602) in PBS with 0.05% Tween 20 (PBST) and incubated with primary antibodies (Supplementary material). After washing with PBST, membranes were incubated with respective horseradish peroxidase-conjugated secondary antibodies (1 h) (Supplementary material), for detection with a Chemiluminescent kit (Novex ECL, Invitrogen, WP20005) and a ChemiDoc MP Imaging system (Bio-Rad). Membrane Coomassie (Sigma-Aldrich, 27816) staining was used for loading control rather than ‘housekeeping’ proteins, as their levels may change depending on tissues analysed and age of the specimen [37]. Densitometric analyses were performed with the Image J software, in non-saturated full dynamic range images. In each independent experiment, samples from control (0 µM GSK) and 10 µM GSK- treated zebrafish were run in the same gel as shown in the supplementary material. Intensity values for each protein were calibrated to the loading control of the same independent experiment.

### Bioinformatic analyses

#### Comparison of PERK orthologues

Protein sequence data from zebrafish (*Danio rerio*, XP_005156642.2), human (*Homo sapiens*, AAI26357.1), mouse (*Mus musculus*, AAH54809.1), and fruit fly (*Drosophila melanogaster*, AAF61200.1) were obtained from UniProt (https://www.uniprot.org/, 28th February 2025). The amino acid sequences from the total human PERK protein and from its kinase domain were compared with its orthologue proteins from zebrafish, mouse, and fruit fly, using the Protein BLAST tool (https://blast.ncbi.nlm.nih.gov/Blast.cgi). Additionally, we determined the identity of the vital amino acids for GSK2606414 binding to the catalytic site of PERK by aligning the protein sequence from zebrafish and human.

#### Zebrafish PERK 3D structure and molecular docking

The predicted three-dimensional protein structures of zebrafish PERK (UniProt: B8JI14) and human PERK (UniProt: Q9NZJ5) were obtained from AlphaFold Protein Structure Database [38, 39]. UCSF ChimeraX software was used to visualize, align (Matchmaker tool), and calculate the root-mean-square deviation (RMSD) of the three-dimensional structures of human PERK (PDB: 4g31) and zebrafish PERK (UniProt: B8JI14), and GSK2606414.

### Statistical analyses

Data are shown as mean ± standard error of the mean (SEM) of the *n* specified in figure legends. For experiments with only two groups (GSK 0 vs. 10 µM), comparisons were made with a *t*-test. For single-factor (e.g., treatment) comparisons of multiple groups, we used one-way ANOVA. For two-factor (e.g., treatment vs. time) analysis, we used two-way ANOVA. For comparing multiple treatment groups against a control group, we used Dunnett’s *post-hoc* test. For conducting all pairwise comparisons among multiple groups, we used Sidak’s *post-hoc* test. For survival analysis, we used the Mantel-Cox (Log-rank) test. *p* < 0.05 was taken as statistically significant. Data analyses were performed with Prism 6.0 (GraphPad).

## Results

### The PERK-GSK2606414 binding site is conserved in humans and zebrafish

To assess the potential of zebrafish to study PERK function and its pharmacological modulation, we performed bioinformatic comparisons of the amino acid and structural identity of zebrafish and human PERK, including specifically the binding site for the PERK inhibitor GSK2606414. We selected GSK2606414 (hereafter GSK; not to be confused with glycogen synthase kinase) because it is one of the most used PERK inhibitors [1]; it was previously used in zebrafish [15]; and the vital amino acids for its binding to PERK are identified [40, 41].

**Fig. 1:**
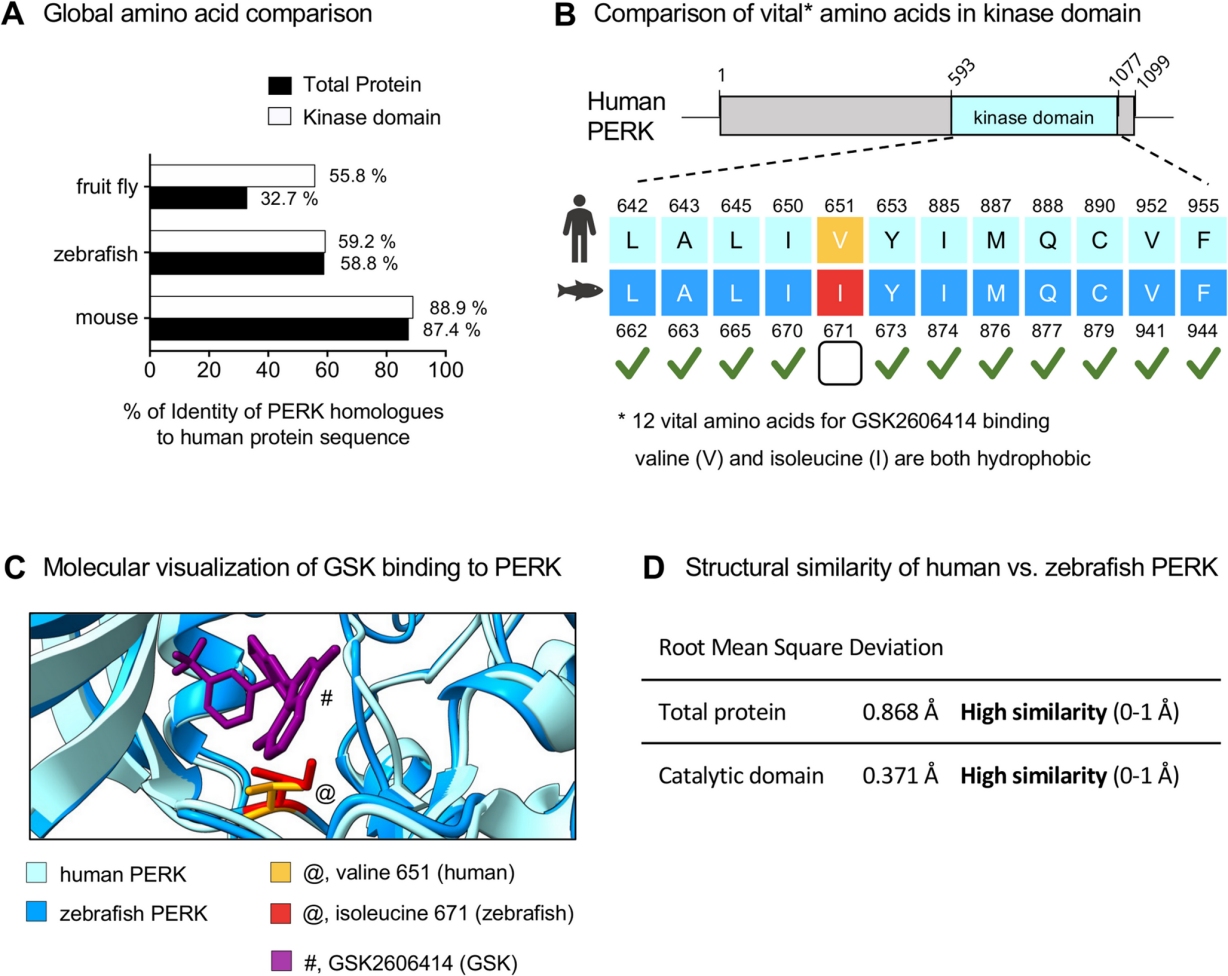
Bioinformatic analyses of PERK (Protein Kinase R-like ER kinase) identity and GSK2606414 (GSK)-binding in human vs. zebrafish. (**A**) Percentage of identity between human, fruit fly (*Drosophila melanogaster*), zebrafish (*Danio rerio*), and mouse (*Mus musculus*) PERK (total and kinase domain); Protein BLAST. (**B**) Comparing vital GSK-binding amino acids in the catalytic domain of human and zebrafish PERK. Valine 651 in human (yellow) is substituted by the also hydrophobic Isoleucine 671 (red) in zebrafish; Protein BLAST. (**C**) Predicted GSK (purple, #) binding to human (light blue) and zebrafish (dark blue) PERK; ChimeraX. (**D**) Root mean square deviation (RMSD) comparing human and zebrafish PERK tertiary structures and respective reference values; ChimeraX

The zebrafish PERK homologue presented 59% global amino acid identity with human PERK (Fig. 1A). Using Protein BLAST, we observed that out of the 12 vital amino acids for GSK binding to PERK [40, 41], there is only one difference between human and zebrafish (Fig. 1B). Such a difference is a valine in humans vs. isoleucine in zebrafish, and both amino acids are hydrophobic. Using molecular visualization with ChimeraX, we observed that the average distance between the 3D structures of zebrafish and human PERK is under 1 Å (Fig. 1C, D), indicating high similarity [42]. The outcome of the statistical analysis is provided in the Figure captions.

These results predict a high probability of GSK binding and inhibiting zebrafish PERK. To test such a prediction, we proceeded with functional assays.

### The PERK inhibitor GSK2606414 is bioactive in zebrafish

**Fig. 2:**
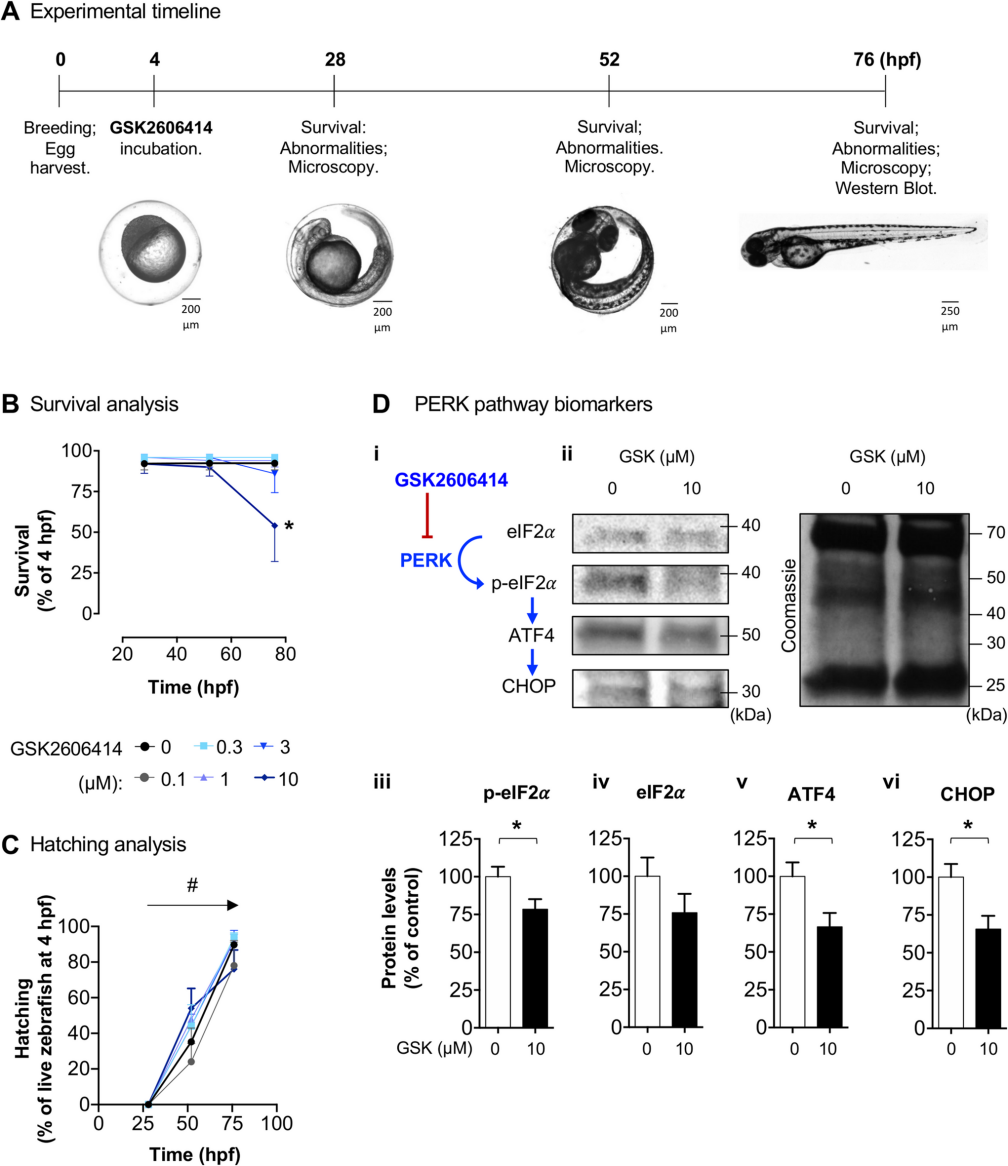
GSK2606414 (GSK) effects on zebrafish survival, hatching, and PERK (Protein kinase R-like ER kinase) pathway biomarkers. (**A**) Experimental timeline. (**B**) Zebrafish survival when exposed to GSK (0–10 µM) over time (4–76 h post-fertilization; hpf); *n* = 50–234 zebrafish, 5 independent breedings, Mantel-Cox test for survival, *p < 0.0001 vs. 0 µM GSK (control). (**C**) Hatching of zebrafish treated with GSK; *n* = 50–234 zebrafish, 5 breedings, two-way ANOVA, treatment (*F*_5,72_ = 1.331, *p* = 0.2609), time (*F*_2,72_ = 227.4, ^#^*p* < 0.0001), treatment ⋅ time interaction (*F*_10,72_ = 1.068, *p* = 0.3981). (**D**) Western blot of live 76 hpf zebrafish: *i*) Schematic representation of the PERK pathway biomarkers; *ii-vi*) Representative blots and quantifications; *n* = 4 independent experiments (pool of 24–27 larvae per condition and per experiment); *t*-test: *iii*) t_6_ = 2.306, *p = 0.0303; *iv*) t_6_ = 1.365, *p* = 0.1107; *v*) t_6_ = 2.572, *p = 0.0422; *vi*) t_6_ = 2.783, *p = 0.0319

To evaluate the effects of PERK inhibition, we evaluated the survival and hatching of zebrafish treated with increasing concentrations of GSK over time (Fig. 2A). We observed a time-dependent decrease in survival, with 10 µM GSK being the concentration that induces a significant effect (50%) at 76 hpf (Fig. 2B, detailed statistical reports are provided in figure captions). For zebrafish that survived GSK, hatching increased time-dependently, without significant differences across GSK concentrations (Fig. 2C). Next, we assessed the effect of GSK on the levels of PERK pathway (ISR) biomarkers in the zebrafish at 76 hpf using western blot (Fig. 2D *i*). Specific protein band selection in zebrafish was assisted by running in the same gel positive control samples (PC12 cells with and without treatment with the ISR activator, thapsigargin) labelled with the same antibodies (Supplementary material). Treatment with GSK (10 µM, 72 h) significantly decreased levels of phosphorylated eIF2α, ATF4, and CHOP (Fig. 2D *iii*, *v*, *vi*). Together, GSK effects confirm its bioactivity and are compatible with PERK pathway inhibition as previously reported [15].

### The PERK inhibitor induces morphological defects in zebrafish

**Fig. 3:**
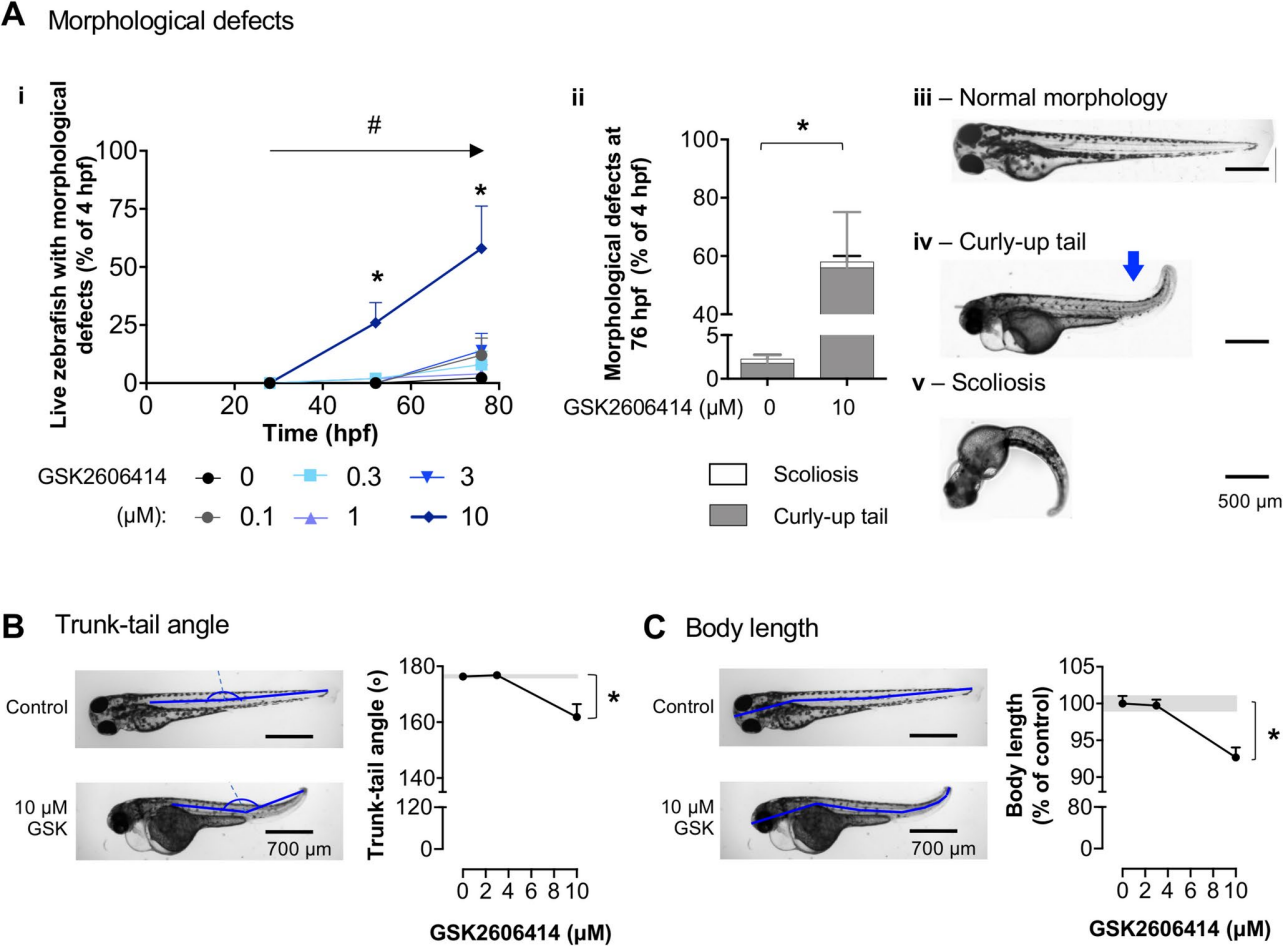
GSK2606414 (GSK) induced morphological defects in zebrafish. (**A**) (*i*) Morphological defects following GSK (0–10 µM) treatment overtime; two-way ANOVA, treatment (*F*_5, 72_ = 9.938, *p* < 0.0001), time (*F*_2, 72_ = 13.14, #*p* < 0.0001), treatment × time interaction (*F*_10,72_ = 3.468, *p* = 0.0009) followed by Dunnett’s multiple comparisons test (*p < 0.05 for 0 vs. 10 µM at 52 hpf or 76 hpf); *n* = 50–234 zebrafish; 5 independent experiments. (*ii-v*) Proportion of curly-up tail and scoliosis at 76 hpf, and representative images; *t*-test (*t*_*8*_ = 3.045, *p = 0.0159); *n* = 50–234 zebrafish; 5 independent experiments. (**B**,** C**) Measurements (blue lines) and quantifications of trunk-tail angle (B), one-way ANOVA (*F*_2, 42_ = 9.882, *p =* 0.0003) followed by Dunnett’s post-hoc (*p < 0.05 for 0 vs. 10 µM) and body length (C), one-way ANOVA (*F*_2, 42_ = 14.57, *p* < 0.0001) followed by Dunnett’s post-hoc (*p < 0.05 for 0 vs. 10 µM) at 76 hpf; *n* = 15 zebrafish, 3 independent experiments

PERK loss-of-function elicits skeletal dysplasia in patients [5]. To investigate the role of PERK in skeletal development, we exposed zebrafish embryos to the PERK inhibitor from 4 to 76 hpf and monitored their development over time. GSK (10 µM) evoked a significant time-dependent increase in morphological defects (Fig. 3A *i*, detailed statistical reports are provided in figure captions). The most common defect was a curly-up tail and a few cases of scoliosis (Fig. 3A *ii*-*iv*). Exposure to GSK (10 µM) caused a 20 ± 5-degree change in the zebrafish trunk-tail angle at 76 hpf (Fig. 3B) and a significant decrease in body length (Fig. 3C).

**Fig. 4:**
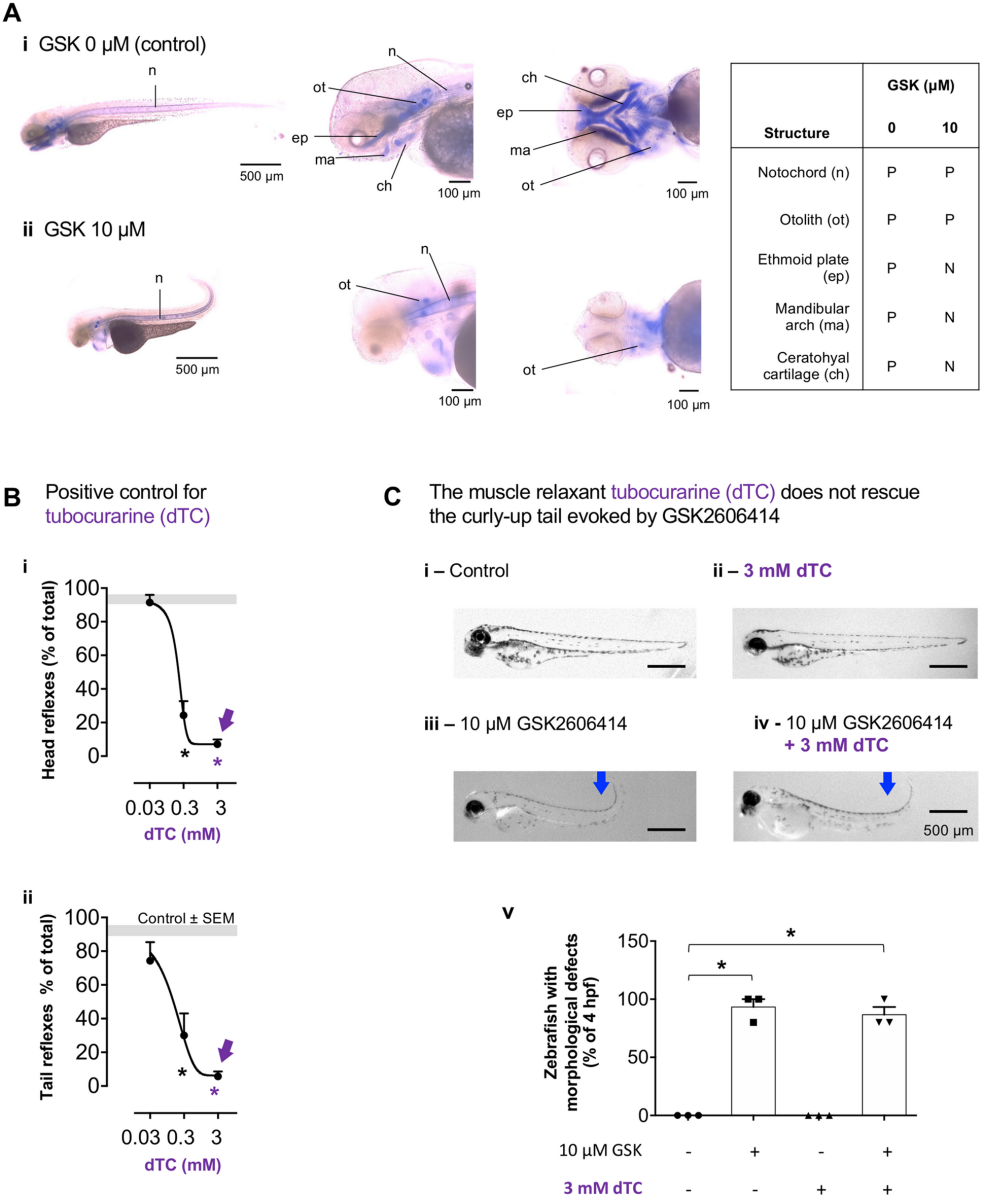
GSK2606414 (GSK) impaired zebrafish skeletal development. **(A)** Representative images of cartilage staining with alcian blue in 76 h post-fertilization (hpf) zebrafish treated with GSK 0 µM (*i*) and 10 µM (*ii*). The table depicts positive (P) or negative (N) labelling for alcian blue in the following skeletal structures: notochord (n), otolith (ot), ethmoid plate (ep), mandibular arch (ma), ceratohyal cartilage (ch). (**B**) Effect of muscle relaxant d-tubocurarine (dTC, 0.03–3 mM) on escape responses after head or tail touch at 76 hpf; Grey bands are mean ± SEM for control zebrafish; one-way ANOVA (*i – F*_3,26_ = 81.37, *p* < 0.0001, *ii* – *F*_3,26_ = 23.35, *p* < 0.0001) followed by Dunnett’s post-hoc (*p < 0.0001 vs. control (0 mM dTC)); *n* = 15 zebrafish, 3 independent experiments. (**C**) (*i-iv*) Representative images of zebrafish for the indicated-treatments. (*v*) Quantification of zebrafish with morphological defects at 76 hpf for the indicated treatments; one-way ANOVA (*F*_1.923, 3.846_ = 146.2, *p* = 0.0012) followed by Sidak’s post-hoc (*p < 0.05 vs. control (0 µM GSK, 0 µM dTC)); *n* = 15 zebrafish; 3 independent breedings. Blue arrows: curly-up tail

To test if the morphological defects evoked by GSK originate in developing skeletal structures, we labelled the cartilage of 3 dpf zebrafish with alcian blue (Fig. 4A). Results show decreased alcian blue staining in the cranium of GSK-treated zebrafish (Fig. 4A *ii*), indicating that PERK inhibition impairs zebrafish skeletal development.

To ascertain if increased muscular contraction contributed to the morphologic defects observed in zebrafish, we tested if the defects were reversed by a muscle relaxant (d-tubocurarine; dTC; antagonist of muscular nicotinic receptors [43]; effective in zebrafish [44]). We observed a concentration-dependent decrease in escape responses, with a maximal relaxation effect at 3 mM (Fig. 4B *i*,* ii*), confirming dTC efficacy as previously described [44]. 3 mM dTC did not recover trunk-tail angle in GSK-treated zebrafish (Fig. 4C), indicating that abnormal skeletal development, rather than muscle contraction, is the main cause of the morphological defects.

We measured otolith area and eye/body ratio (Supplementary material) as negative controls (unrelated to WRS phenotypes) and found no changes upon GSK exposure, indicating that GSK effects on zebrafish morphology are selective.

These results indicate that the PERK inhibitor evoked abnormal skeletal development and growth retardation in zebrafish, indicating that this small vertebrate holds potential to study the role of PERK in skeletal development.

### The PERK inhibitor delays endocrine pancreas development and limits glucose uptake in zebrafish

Lack of PERK in WRS patients leads to decreased 𝛽-cell mass, impaired insulin production and neonatal diabetes [45, 46]. Exocrine pancreas dysfunction was found in only 10.6% of patients [47, 48]. Thus, we explored if zebrafish could be useful to study PERK’s role in pancreatic development.

**Fig. 5:**
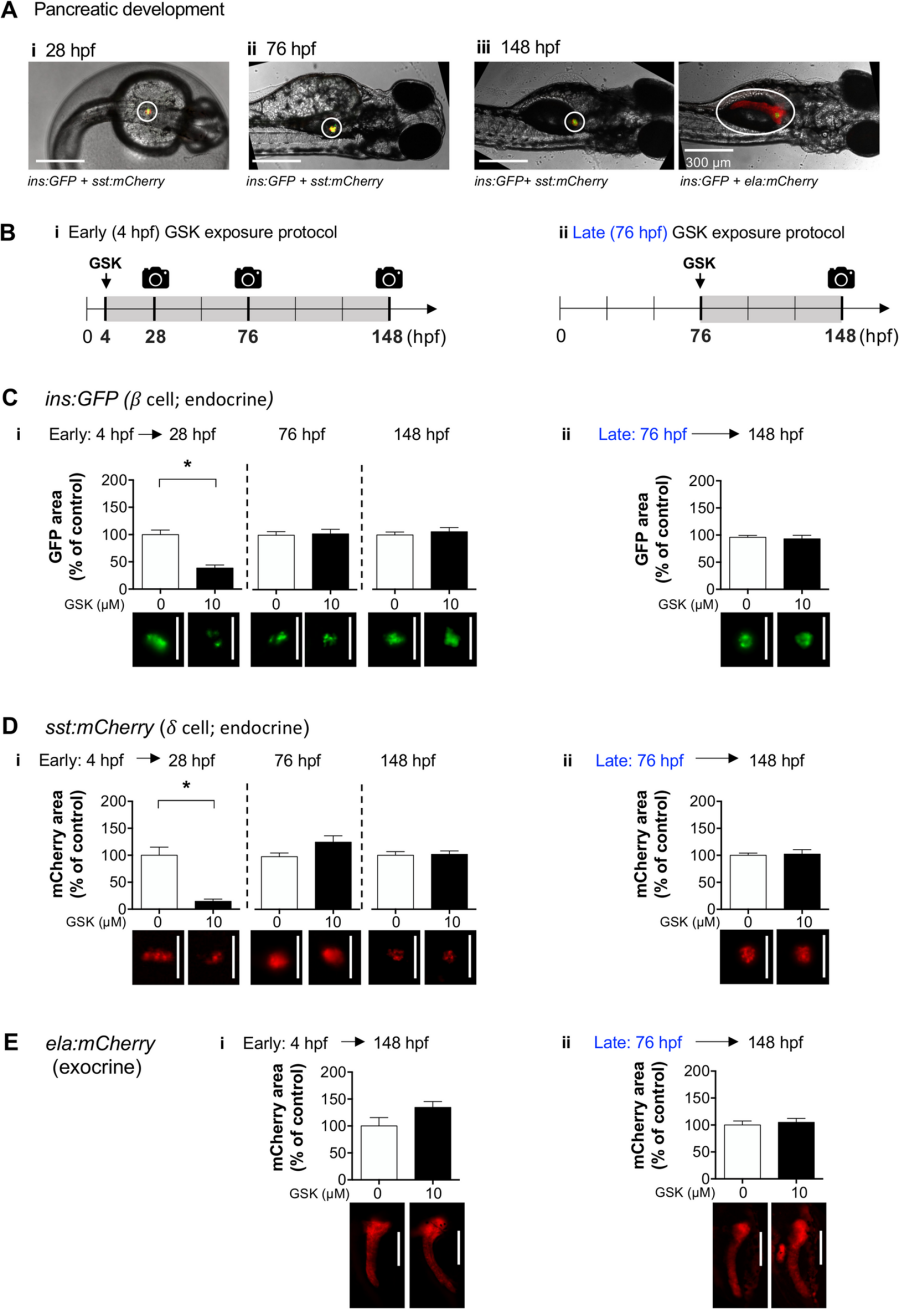
Pancreatic development in transgenic zebrafish. **(A)** Representative images of normal pancreas development in Tg(*ins*:*GFP*, *sst*:*mCherry*) and Tg(*ins*:*GFP*, *ela*:*mCherry*) zebrafish. (**B)** early (*i*) and late (*ii*) GSK2606414 (GSK)-exposure protocols. (**C-E**) Area measurements for *ins:GFP* (green, insulin-producing 𝛽-cells), *sst: mCherry* (red, somatostatin-producing 𝛿-cells), and *ela:mCherry* (red, elastase-producing exocrine cells), in zebrafish exposed to 10 µM GSK since 4 h post-fertilization (hpf) (*i*, early exposure) or since 76 hpf (*ii*, late exposure); *t*-test, (C*i* – *t*_25_ = 2.503, *p = 0.019, D*i* – *t*_13_ = 3.022, *p = 0.0098); *n* = 4–33 larvae. Representative images of GFP and mCherry are shown below the respective bars. Scale bars indicate 200 μm

To ascertain the effects of PERK inhibition in exocrine and endocrine pancreas development, we used two transgenic zebrafish lines expressing GFP in insulin-producing *β*-cells (Fig. 5A). one line also expressed mCherry in somatostatin-producing 𝛿-cells from the endocrine pancreas – Tg(*ins:GFP*,* sst:mCherry*); the other line expressed mCherry in elastase-producing exocrine pancreas –Tg(*insGFP*,* ela:mCherry*). We exposed both lines to GSK (10 µM) and measured the GFP/mCherry labelled areas as proxies for cell mass.

To test if the effects of the PERK inhibitor were selective for the early embryo development, we used two exposure protocols: the *early exposure protocol* (Fig. 5B *i*) started before the formation of the first Langerhans islet (endocrine pancreas); the *late exposure protocol* started after islet formation (Fig. 5B *ii*). Early exposure (4 hpf) delayed development of the endocrine pancreas, significantly decreasing *β* and 𝛿 cell mass at 28 hpf, which seems to recover over time (Fig. 5C *i*, Fig. 5D *i*, detailed statistical reports are provided in figure captions). Late exposure (76 hpf) failed to alter endocrine cell mass (Fig. 5C *ii*, Fig. 5D *ii*). Regarding the exocrine pancreas mass, *ela:mCherry* fluorescence was only detectable at 148 hpf, and remained unchanged with either early or late GSK exposure protocols (Fig. 5E). These findings support the hypothesis that PERK inhibition delays the early embryonic development of the endocrine pancreas.

**Fig. 6:**
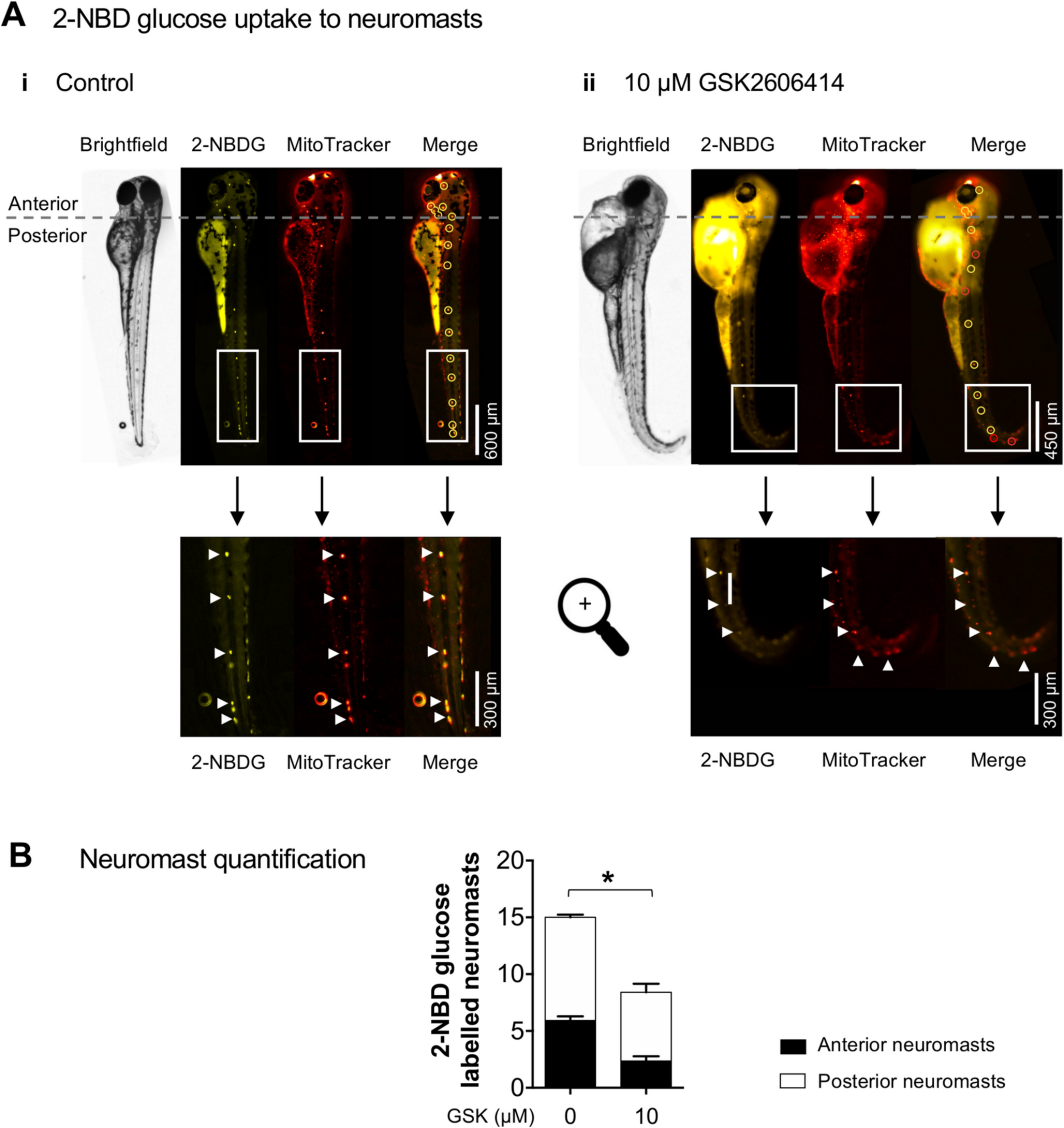
GSK2606414 (GSK) effects on 2-NBDglucose (2-NBDG) uptake in zebrafish. (**A**) Representative brightfield and fluorescence images of zebrafish at 76 h post-fertilization (hpf), following treatment with (*i*) 0 µM (control) or (*ii*) 10 µM GSK since 4 hpf. Images show functional labelling at 76 hpf with the fluorescent glucose analogue 2-NBDG for neuromasts that uptake glucose, and with MitoTracker Deep Red that labels mitochondria in neuromasts as a positive control. In the “merge” images, yellow circles highlight neuromasts with both 2-NBDG and MitoTracker, while red circles highlight those containing MitoTracker only. Below, 2× amplified images show neuromasts from the zebrafish lateral line, with arrows indicating neuromasts on the side proximal to the microscope objective (the paired neuromasts on the opposite side are unmarked). (**B**) Quantification of neuromasts labelled with 2-NBDglucose; *t*-test (*t*_29_ = 5.069, *p < 0.0001); *n* = 9–12 zebrafish per condition

To test if delayed endocrine development affected glucose homeostasis, we measured the uptake of the fluorescent glucose probe 2-NBDglucose [31] in zebrafish treated with GSK from 4 to 76 hpf (early exposure protocol). 2-NBDglucose uptake was measured in neuromast cells, given their direct exposure to probes in the external media, using MitoTracker Deep Red as a positive control for neuromast staining. Early exposure to the PERK inhibitor significantly decreased the uptake of 2-NBDglucose (Fig. 6A, B), indicating that PERK inhibition induces a diabetic-like phenotype in zebrafish.

### The PERK inhibitor evokes neuromuscular and cardiac impairments in zebrafish

**Fig. 7:**
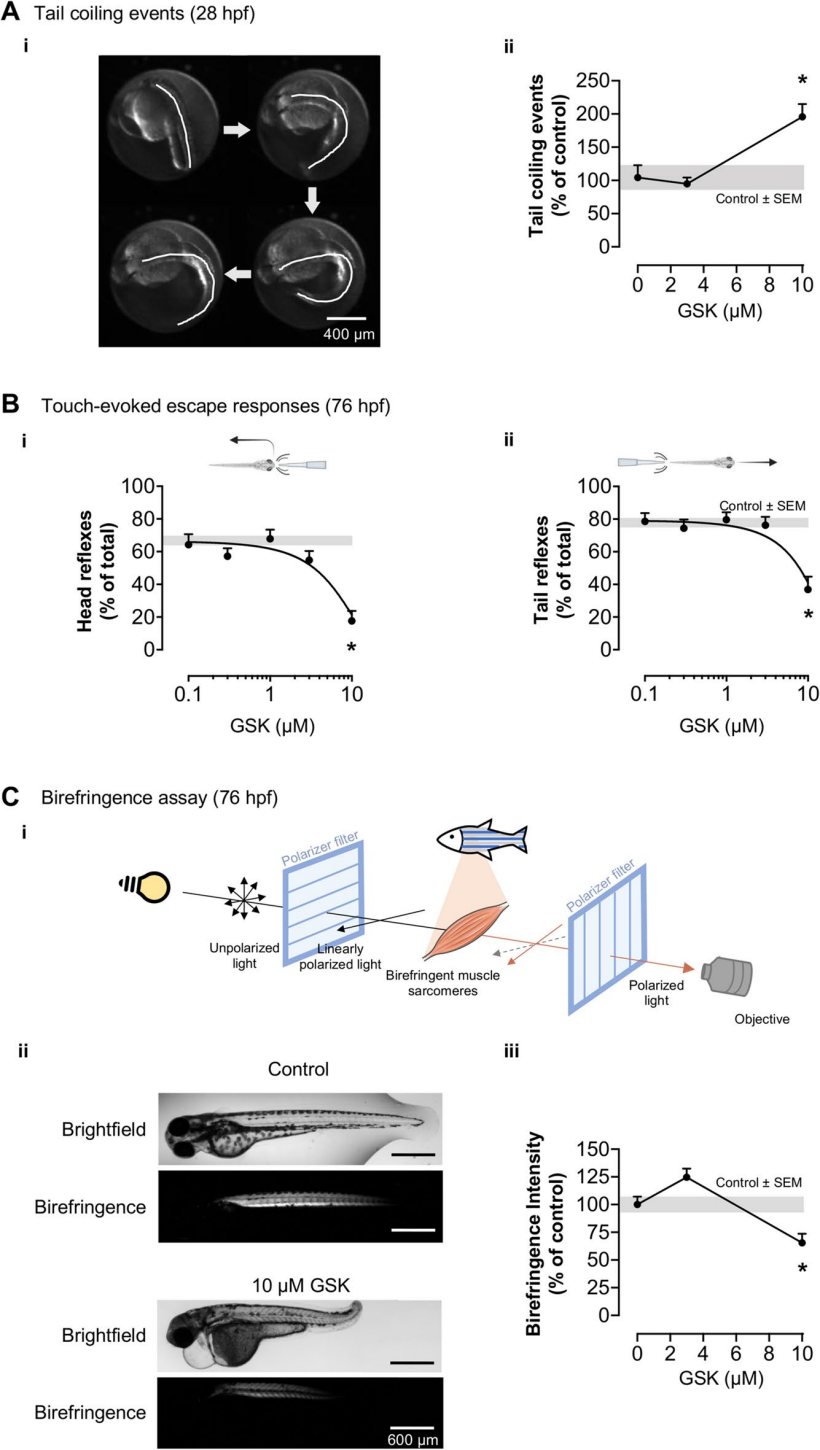
GSK2606414 (GSK) effects on neuromotor responses and muscle integrity. (**A**) (*i*) Representative tail coiling time-lapse: white line highlights notochord movement. (*ii*) Quantification of tail coiling in 28 h post-fertilization (hpf) zebrafish treated with GSK since 4 hpf; one-way ANOVA (*F*_2,70_ = 11.69, *p* < 0.0001) followed by Dunnett’s post-hoc (*p = 0.0004 vs. control (0 µM GSK)); *n* = 23–26 zebrafish. (**B**) Quantification of escape responses after head (*i*) or tail (*ii*) touches at 76 hpf; one-way ANOVA (*i* – *F*_5,240_ = 6.623, *p* < 0.0001; *ii* – *F*_5,230_ = 5.087, *p* = 0.0002) followed by Dunnett’s post-hoc (*p < 0.0001 vs. control (0 µM GSK)); *n* = 13–114 zebrafish. (**C**) (*i*) Schematic representation of the birefringence assay for muscle integrity; (*ii*) Representative images of muscle birefringence at 76 hpf. (*iii*) Quantification of birefringence intensity; one-way ANOVA (*F*_2,42_ = 14.77, *p* < 0.0001) followed by Dunnett’s post-hoc (*p = 0.0057 vs. control (0 µM GSK)); *n* = 15 zebrafish (**A-C**) Grey bands are mean ± SEM for 0 µM GSK (control)

Beyond diabetes and skeletal dysplasia, patients with loss-of-function mutations in PERK may also present impaired brain development [49], muscle weakness [50], and cardiac alterations [51, 52]. We first assessed the role of PERK on zebrafish neuromuscular function by measuring spontaneous tail-coiling and touch-evoked escape responses upon exposure to the PERK inhibitor [53–55]. GSK (10 µM) significantly increased tail-coiling events in early development (28 hpf embryos inside the chorion; Fig. 7A, detailed statistical reports are provided in figure captions) and significantly decreased escape responses in later development (76 hpf larvae; Fig. 7B *i*, *ii*). To test for altered muscle integrity, we assessed the arrangement of sarcomeres by measuring how well they diffracted polarized light (birefringence assay) [56] (Fig. 7C *i*). Treatment with 10 µM GSK significantly decreased muscle birefringence intensity (Fig. 7C *ii*,* iii*).

**Fig. 8:**
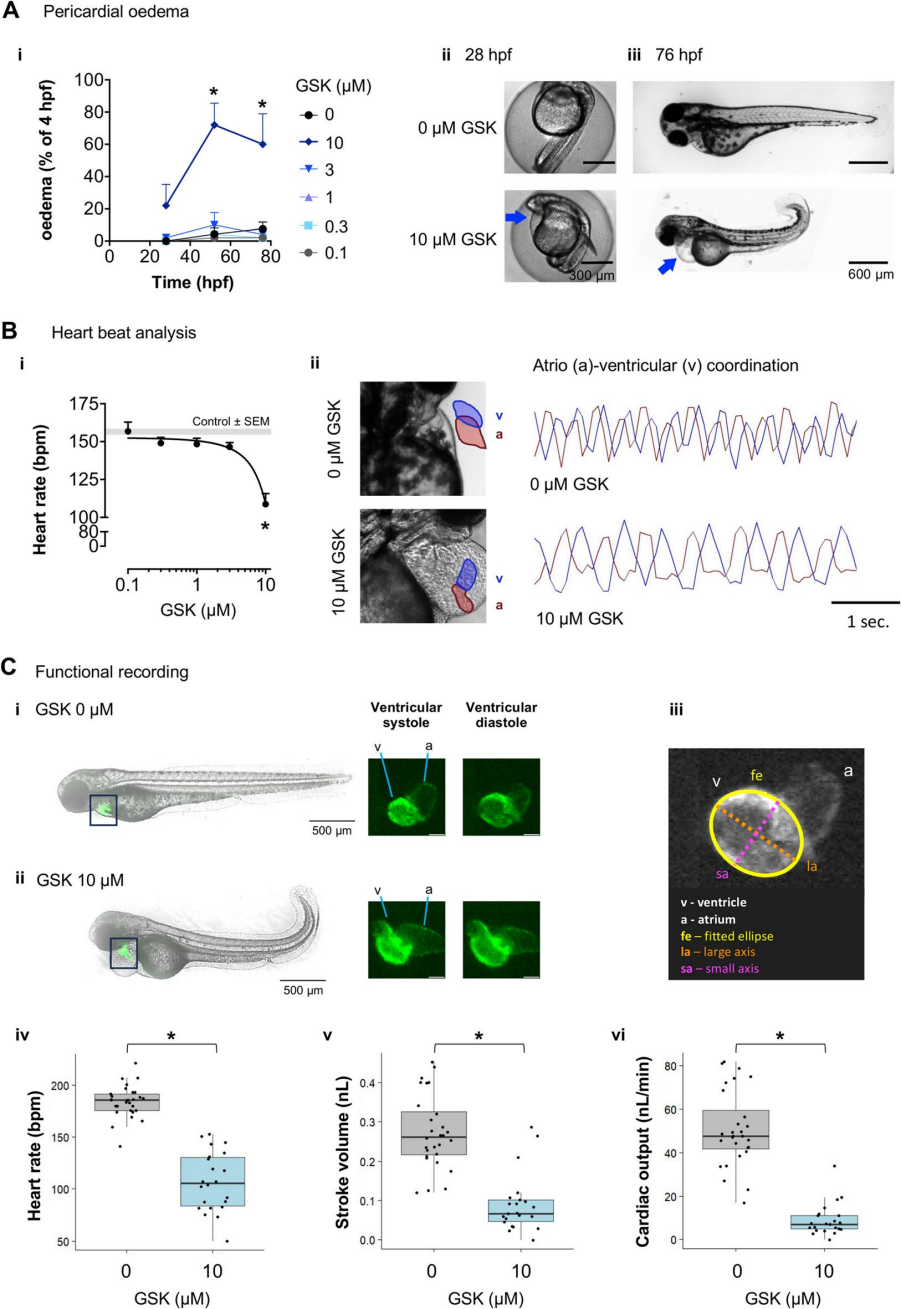
GSK2606414 (GSK)-induced morphological and functional cardiac defects. (**A**) (*i*) Quantification of live zebrafish with pericardial oedema at the indicated timepoint (hours post-fertilisation, hpf) and GSK concentrations; two-way ANOVA, treatment (*F*_5,72_ = 23.96, *p* < 0.0001), time (*F*_2,72_ = 5.124, *p* = 0.0083), treatment ⋅ time interaction (*F*_10,72_ = 2.010, *p* = 0.045) followed by Dunnett’s multiple comparisons test (* *p* < 0.0001 for 0 vs. 10 µM at 52 or 76 hpf); *n* = 50–234 zebrafish, 5 independent experiments. (*ii*,* iii*) Representative images of zebrafish treated with GSK vs. control; blue arrow: pericardial oedema. (**B**) (*i*) Heartbeats per minute (bpm) of 76 hpf zebrafish exposed to increasing GSK concentrations since 4 hpf; grey band is mean ± SEM for control; one-way ANOVA (*F*_5,243_ = 11.08, *p* < 0.0001) followed by Dunnett’s post-hoc, *p < 0.0001 vs. control; *n* = 13–116 zebrafish; (*ii*) Representative images of the heart and plots of atrio-ventricular coordination in control and 10 µM GSK-treated zebrafish (a: atrium – red; v: ventricle – blue). **(C)** Cardiac function recording of Tg(*hsp70l*: *DsRed2*, *cmlc2*:*EGFP*) zebrafish treated with 0 µM GSK (*i*) or 10 µM GSK (*ii*). (*iii*) By fitting an ellipse (fe - yellow) to the cross-section of the heart ventricle, imaged along the sagittal plane, we measured its large (major) axis (la - orange) and small (minor) axis (sa - pink). (*iv*) Heartbeat measurement; *t*-test (*t*_30.625_ = 11.435, *p < 0.0001). The large and small axes measurements were used to calculate (*v*) stroke volume; *t*-test on log-transformed data (*t*_28.379_ = 7.5574, *p < 0.0001) and (*vi*) cardiac output; *t*-test on log-transformed data (*t*_30.23_ = 11.635, *p < 0.0001), *n* = 22–28 zebrafish per condition, from 3 independent breedings

Next, we monitored cardiac morphology and function in PERK-inhibited zebrafish, where 10 µM GSK induced a time-dependent increase in pericardial oedema (Fig. 8A). At 76 hpf, 10 µM GSK induced bradycardia (Fig. 8B, C *iv*), and reduced stroke volume (Fig. 8C *v*) and cardiac output (Fig. 8C *vi*) without altering atrio-ventricular coordination in zebrafish (Fig. 8B), supporting the hypothesis that PERK inhibition impairs cardiac morphology and function.

These results show that the PERK inhibitor GSK2606414 evokes neuromuscular and cardiac abnormalities in morphology and function in zebrafish, indicating that this small vertebrate is a suitable model to study PERK effects on neuromuscular and cardiac development and function.

## Discussion

In the present study we show that the PERK inhibitor GSK2606414 causes defective skeletal development and diabetic-like phenotypes, cardiac and neuromuscular alterations in zebrafish, mirroring key features of Wolcott-Rallison syndrome patients. These results highlight the potential of zebrafish to study PERK’s role in developmental diseases with multisystem involvement, such as the WRS.

The PERK inhibitor GSK2606414, one of the most used PERK inhibitors in the scientific literature, decreased ISR activation in animals like mice [57], frogs [58], and flies [15]. In the present study, we show that GSK2606414 decreases the levels of ISR markers in zebrafish, which suggests that GSK2606414 is bioactive and effective in decreasing PERK pathway activity. Additionally, GSK2606414 evoked key WRS-associated phenotypes (impaired skeletal development and diabetic-like phenotype), while sparing non-WRS-associated parameters, such as otolith area and eye/body ratio. Although we cannot exclude GSK2606414 off-targeting c-KIT and RIPK (receptor-interacting serine/threonine-protein kinase 1) kinases [59, 60], zebrafish with genetically inhibited KITa/b and RIPK3 [61, 62] did not show the curly-up tail and other structural alterations reported in our study, suggesting that such alterations are not off-target effects of GSK2606414 in c-KIT and RIPK kinases. Thus, these results are compatible with GSK2606414 selectively inhibiting PERK in zebrafish.

Loss of PERK function causes growth retardation and skeletal dysplasia in humans [5] and mice, via impairments in collagen secretion, osteoblast differentiation and bone mineralization [9, 63, 64]. Here, we show that the PERK inhibitor GSK2606414 in zebrafish causes growth retardation, spinal deformities resistant to muscle relaxation with tubocurarine, and decreased cartilage staining. Since at the time of the assays (76 hpf), the zebrafish cartilage is starting to ossify [65], our findings indicate that the PERK inhibitor GSK2606414 impairs zebrafish skeletal development.

PERK controls pancreatic 𝛽-cell development and insulin production [66]. Loss of PERK function in humans and mice causes decreased pancreatic mass and neonatal diabetes. PERK ablation studies in mice indicate that impaired 𝛽-cell proliferation, rather than increased cell death, explain the decreased pancreatic mass, and that defective proinsulin maturation in the endoplasmic reticulum contributes to neonatal diabetes [67–70]. Here we show that exposure to the PERK inhibitor GSK2606414 during early zebrafish development delays endocrine pancreas formation, resulting in a decreased 𝛽-cell mass, which gradually recovers overtime. To assess whether glucose homeostasis is impaired by GSK2606414 in zebrafish, we used the fluorescence glucose uptake probe 2-NBDglucose. We found reduced 2-NBDglucose uptake, which indicates that glucose homeostasis remains partly compromised despite the ongoing 𝛽-cell mass recovery. Such recovery in the presence of GSK2606414 suggests that zebrafish can regenerate pancreatic tissue [71, 72] by PERK-independent mechanisms. The identification of such PERK-independent mechanisms in zebrafish may assist the development of novel therapies for 𝛽-cell regeneration for human diabetes [73].

In contrast to the delayed development of the zebrafish endocrine pancreas, we found no effect of the PERK inhibitor upon the mass of the exocrine pancreas. While different mechanisms regulate the development of the endocrine and exocrine pancreas [47, 69], we cannot exclude that extended exposure of zebrafish to the PERK inhibitor may alter the mass of the exocrine pancreas. Future studies using selective PERK knockout in zebrafish pancreatic cell subtypes may contribute to understanding differential pancreatic vulnerability.

Beyond pancreatic dysfunction, PERK inhibition in humans and mice impairs neuronal and muscular functions [5, 6, 11, 74, 75]. Here we show that the PERK inhibitor increases zebrafish spontaneous tail movements early in development, which arise in a spinal central pattern generator that provides rhythmic depolarizations [53, 55], and decreases touch-evoked escape responses later in development, which arise in sensory neurons [54, 55]. We also show that zebrafish exposed to the PERK inhibitor display altered muscular integrity, likely contributing to decreased escape responses. Thus, as with mammals, the mechanisms underlying motor impairment in PERK-inhibited zebrafish combine dysfunctions in neurons and muscle.

Patients and mice with PERK loss of function often display cardiac malformations [10, 51, 52]. Here we show that PERK-inhibited zebrafish display cardiac abnormalities, such as oedema and bradycardia. The transparency and small size of young zebrafish are amenable to the detection of cardiac abnormalities that would be fatal in larger animals due to insufficient oxygen diffusion [76]. Moreover, zebrafish may assist in the discovery of heart regeneration mechanisms [77]. Thus, future studies with a more detailed structural and functional characterization of the cardiac abnormalities in PERK-inhibited or deleted zebrafish may provide valuable insights into disease mechanisms and potential therapeutics.

Our proof-of-concept study shows that the PERK inhibitor GSK2606414 evokes developmental defects in zebrafish consistent with the WRS. The pharmacological approach used has limitations, and further complementary studies are necessary to discern specific PERK inhibition effects versus pharmacological off-target effects. Future pharmacological studies should include different classes of PERK inhibitors and be complemented with genetic studies. Genetic ablation of PERK would more accurately mimic WRS aetiology in zebrafish and contribute to ascertaining the selectivity of WRS-related phenotypes in PERK-inhibited zebrafish. Previous studies using morpholino knockdown of PERK activity were not aimed at evaluating WRS-related phenotypes [17]. We suggest that future studies employing CRISPR/Cas9 technology can be used to generate zebrafish models with loss of PERK function, as well as with conditional/selective expression of human PERK with WRS-driving mutations [78].

## Conclusions

This study highlights the potential of the small vertebrate zebrafish as a model for developmental diseases, namely those caused by PERK inhibition, like the WRS. We show that the pharmacological inhibitor of PERK, GSK2606414, mimicked WRS pathophysiology by inducing defective growth and skeletal development, and diabetic-like phenotypes such as delayed development of the endocrine pancreas and altered glucose homeostasis. The PERK inhibitor also impaired neuromuscular and cardiac development, while sparing non-WRS-associated parameters like otolith area and eye/body ratio. Key follow-up studies in zebrafish may include the testing of different classes of PERK inhibitors, genetic approaches for PERK silencing, and the conditional/selective expression of human PERK with WRS-driving mutations. Also, future research on the mechanisms governing PERK-independent pancreatic regeneration in zebrafish may uncover potential therapeutic targets for diabetes. Further, the scalability of zebrafish models holds great potential to enhance the efficiency of drug screening against PERK-associated developmental diseases.

## Supplementary Information

Below is the link to the electronic supplementary material.

Supplementary Material 1

Supplementary Material 2

Supplementary Material 3

## Data Availability

The datasets generated during and/or analysed during the current study are available from the corresponding author upon reasonable request.

## Abbreviations

2-NBDglucose: 2-(7-Nitro-2,1,3-benzoxadiazol-4-yl)-D-glucosamine
ATF4: Activating transcription factor 4
CHOP: C/EBP homologous protein
dpf: Days post-fertilization
dTC: d-tubocurarine
eIF2α: Eukaryotic translation initiation factor 2α
ER: Endoplasmic reticulum
hpf: Hours post-fertilization
GSK: GSK2606414
ISR: Integrated stress response
NPH: Nikon-Prior-Hamamatsu
PERK: Protein kinase R-like ER kinase
WRS: Wolcott-Rallison syndrome
