# Supplementary material for: The PERK inhibitor GSK2606414 evokes developmental defects in zebrafish consistent with Wolcott-Rallison syndrome phenotypes
Source: Pharmacol Rep. 2026 Feb 13;78(2):487–504. doi: 10.1007/s43440-026-00837-7 (PMC12975795; doi:10.1007/s43440-026-00837-7)
Supplement: Supplementary file 1 — Supplementary Material 1 [file 43440_2026_837_MOESM1_ESM.pdf]

Supplementary Material

Supplementary Figure 1. Original western blots present in Fig. 2 D *ii*.

Supplementary Figure 2. GSK2606414 (GSK) effect on zebrafish otolith area.

Supplementary Figure 3. GSK2606414 (GSK) effect on zebrafish eye/body ratio.

Supplementary Figure 4. Original p-eIF2α western blots used for quantification of data in Fig. 2 D *iii.*

Supplementary Figure 5. Original eIF2α western blots used for quantification of data in Fig. 2 D *iv.*

Supplementary Figure 6. Original ATF4 western blots used for quantification of data in Fig. 2 D *v.*

Supplementary Figure 7. Original CHOP western blots used for quantification of data in Fig. 2 D *vi*.

Supplementary Figure 8. Original western blot membranes stained with Coomassie used as loading control.

Supplementary Table 1. Primary antibodies used in western blots from Fig. 2 D.

Supplementary Table 2. Secondary antibodies used in western blots from Fig. 2 D.

Supplementary Video 1. Functional cardiac recording of Tg(*hsp70l:DsRed2; cmlc2:EGFP*) zebrafish treated with 0 μM GSK

Supplementary Video 2. Functional cardiac recording of Tg(*hsp70l:DsRed2; cmlc2:EGFP*) zebrafish treated with 10 μM GSK

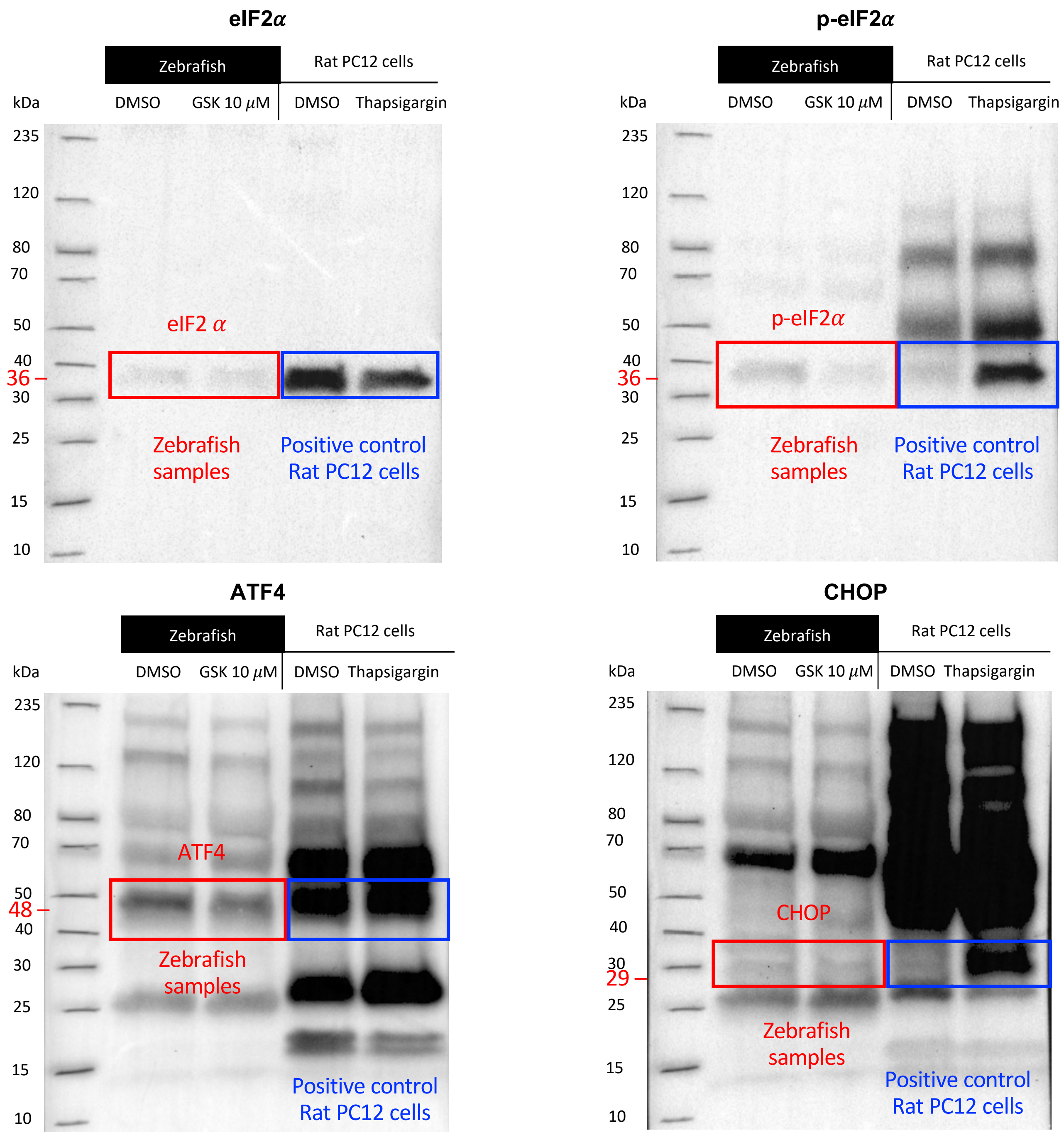

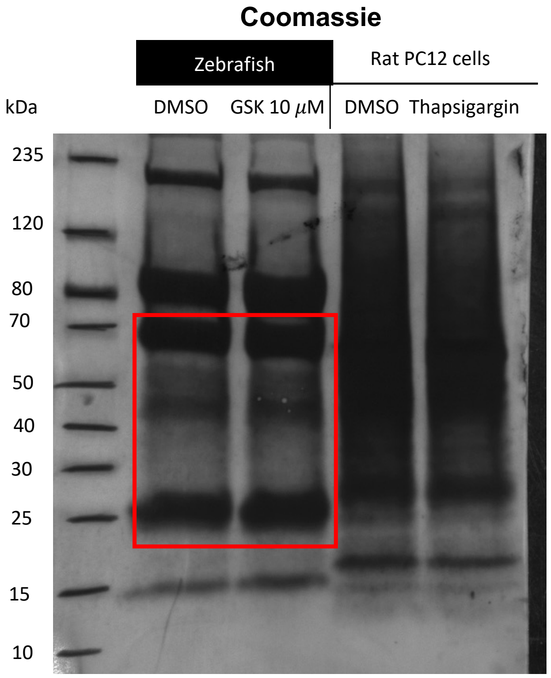

Supplementary Figure 1. Original western blots: red boxes show the cropped western blots present in Fig. 2 D *ii*. Band selection for zebrafish samples was made by comparison with positive control samples of PC12 cells incubated with thapsigargin (Tg), an activator of the integrated stress response[1]. Chosen exposure time was before signal saturation in zebrafish samples.

[1] L.M. Almeida, A. Oliveira, J.M.A. Oliveira, B.R. Pinho, Stress response mechanisms in protein misfolding diseases: Profiling a cellular model of Huntington's disease, Arch Biochem Biophys, 745 (2023) 109711, https://doi.org/10.1016/j.abb.2023.109711.

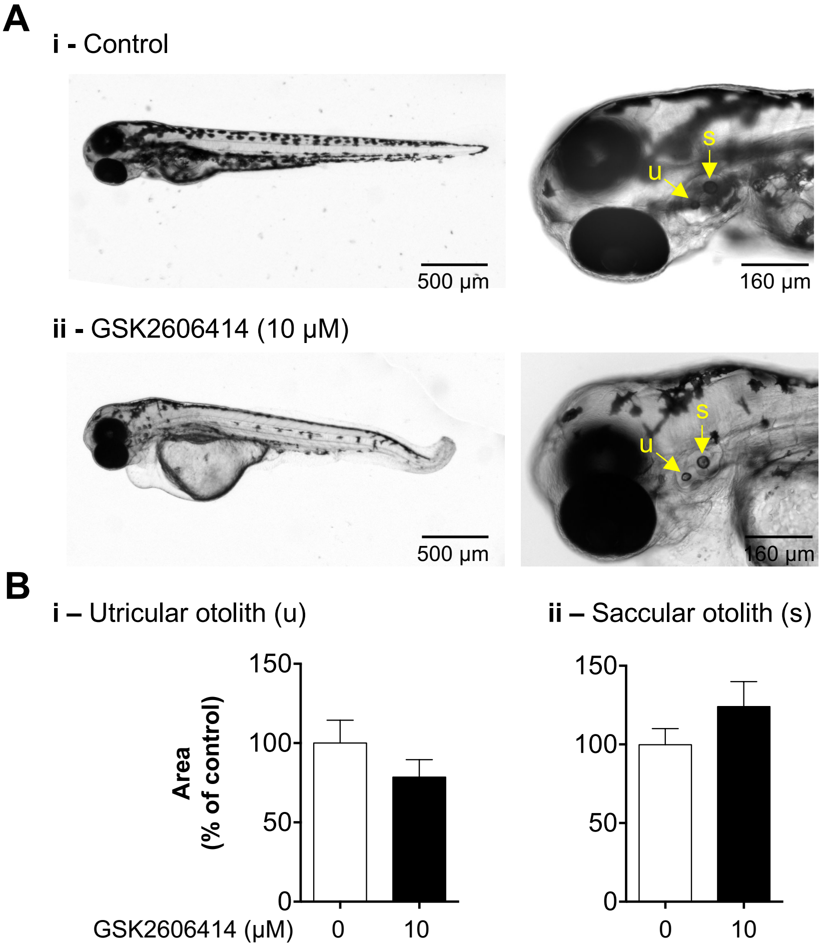

Supplementary Figure 2. GSK2606414 (GSK) does not alter zebrafish otolith area. **(A)** Representative images of zebrafish at 76 hpf following treatment with control (0 µM GSK) or 10 µM GSK since 4 hpf. Arrows: (u) utricular/anterior otolith, (s) saccular/posterior otolith. **(B)** Mean ± SEM of the otolith area, $P > 0.05$, t-test, $n = 15$ larvae, 3 zebrafish experiments.

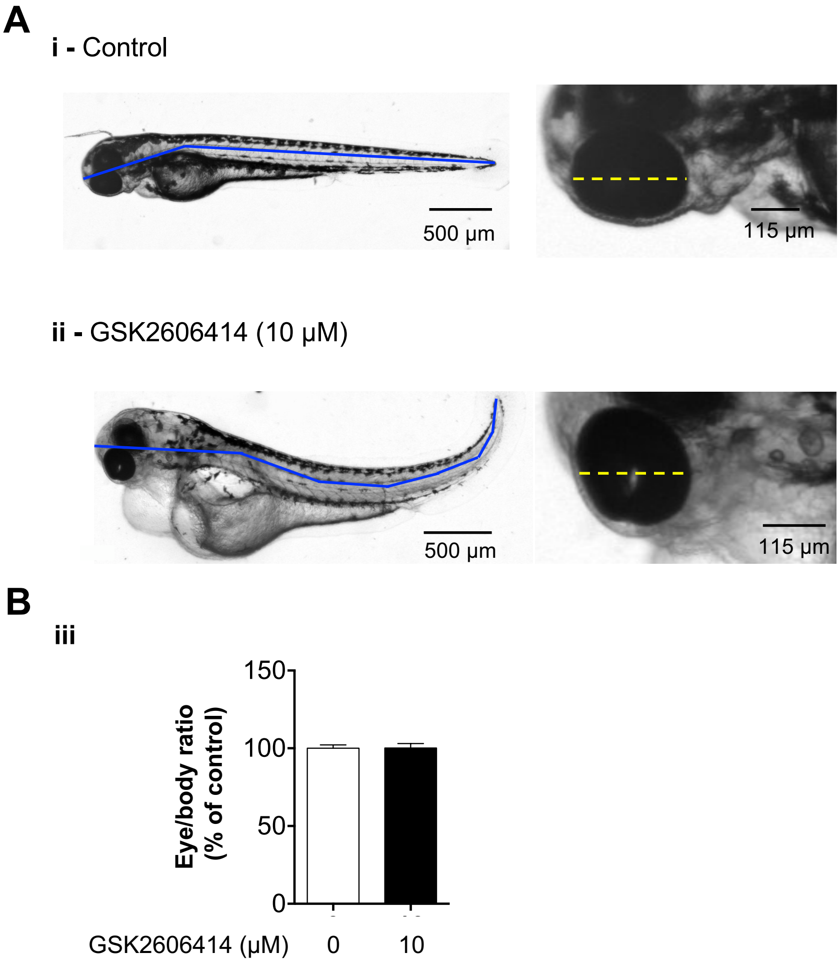

Supplementary Figure 3. GSK2606414 does not alter zebrafish eye/body ratio. **(A)** Representative images of zebrafish at 76 hpf following treatment with control (0 µM GSK) or 10 µM GSK since 4 hpf. Solid blue lines: body length; dashed yellow lines: eye diameter. **(B)** Mean ± SEM of the eye/body ratio, $P > 0.05$, t-test, $n = 15$ zebrafish, 3 independent experiments.

p-eIF2α (expected molecular weight - 36 kDa)

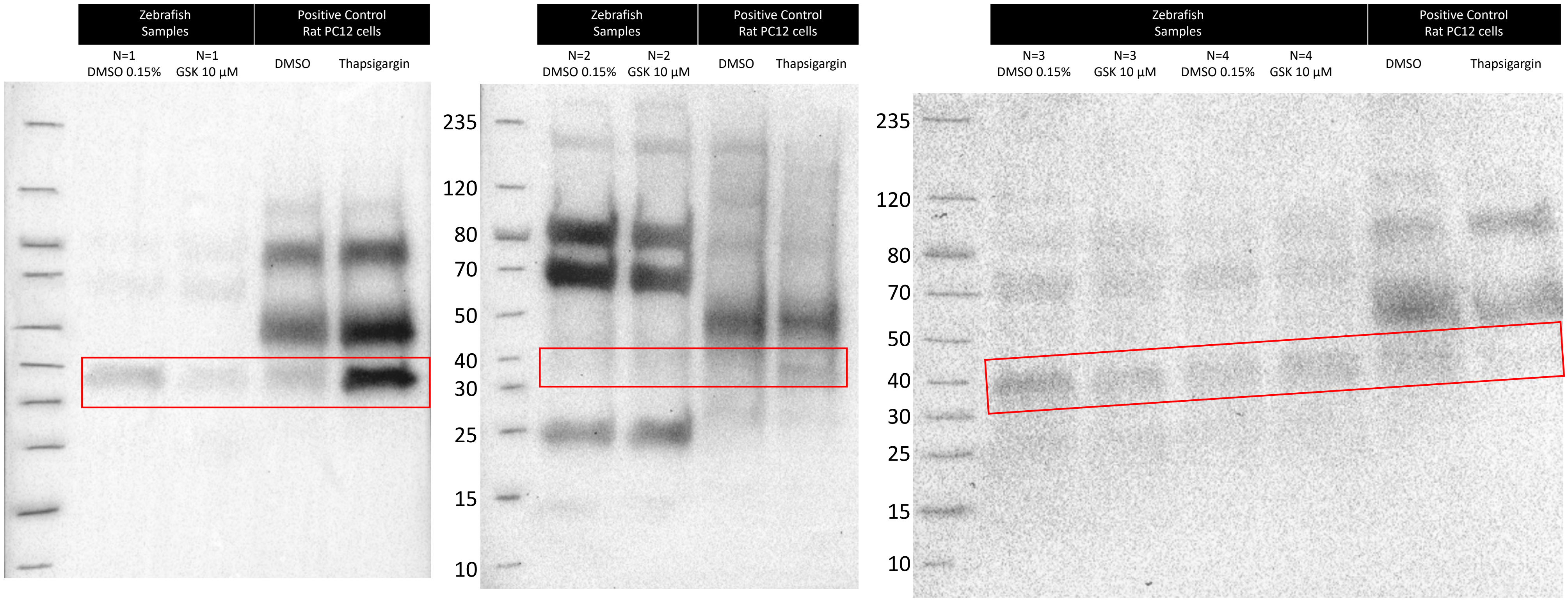

Supplementary Figure 4. Original western blots used for quantification of data in Fig. 2 D *iii*: red boxes show the quantified band. Band selection for zebrafish samples was made by comparison with positive control samples of PC12 cells incubated with thapsigargin (Tg), an activator of the integrated stress response. Chosen exposure time was before signal saturation in zebrafish samples.

eIF2α (expected molecular weight - 36 kDa)

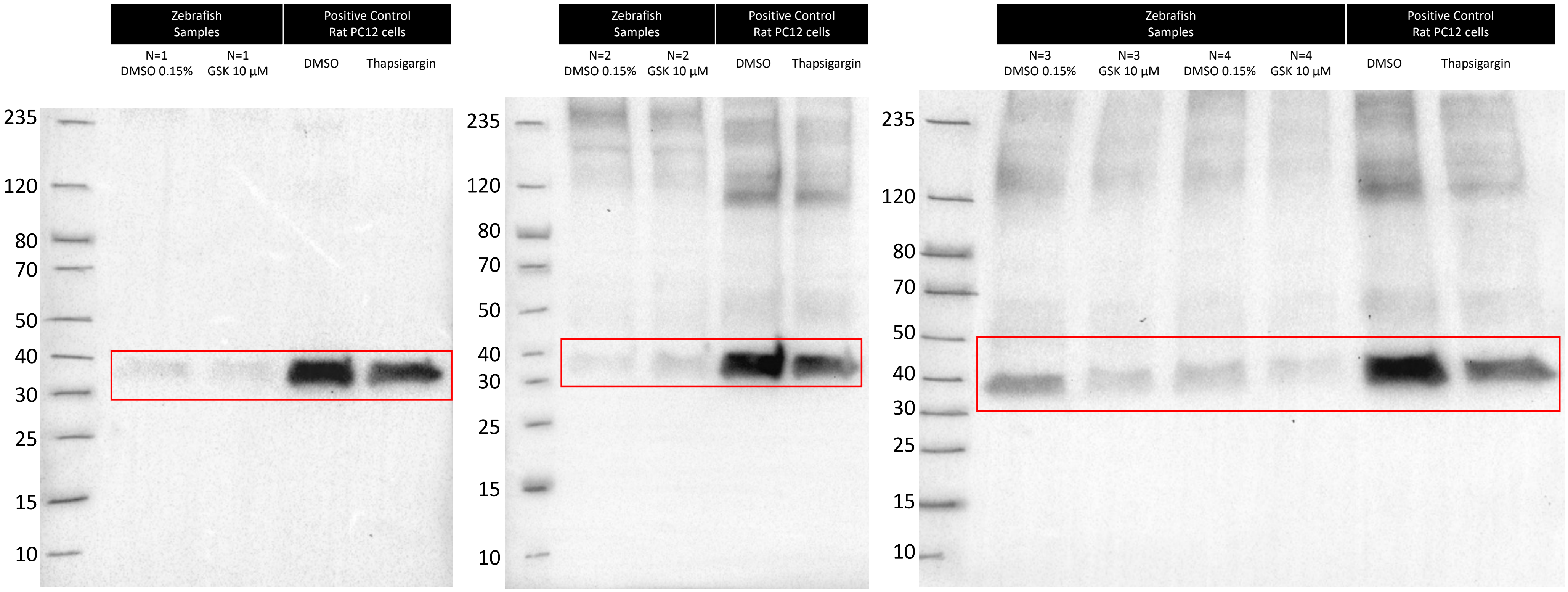

Supplementary Figure 5. Original western blots used for quantification of data in Fig. 2 D *iv*: red boxes show the quantified band. Band selection for zebrafish samples was made by comparison with positive control samples of PC12 cells incubated with thapsigargin (Tg), an activator of the integrated stress response. Chosen exposure time was before signal saturation in zebrafish samples.

ATF4 (expected molecular weight - 48 kDa)

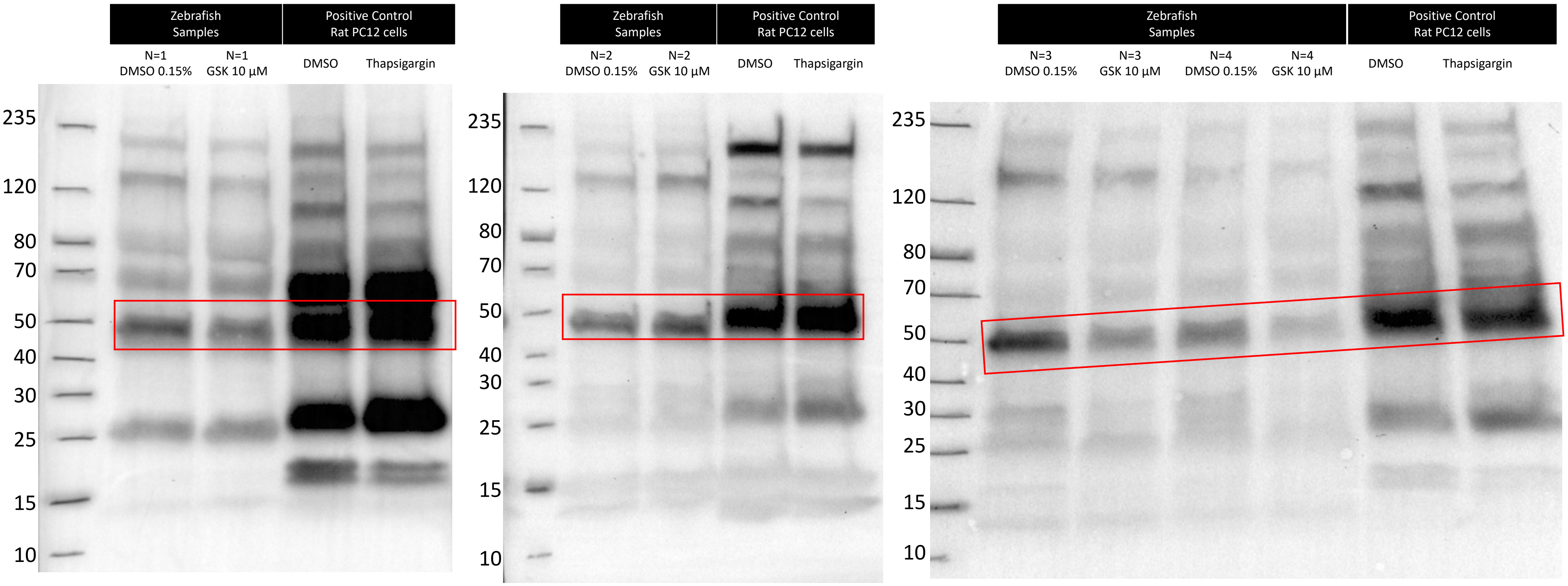

Supplementary Figure 6. Original western blots used for quantification of data in Fig. 2 D *v*: red boxes show the quantified band. Band selection for zebrafish samples was made by comparison with positive control samples of PC12 cells incubated with thapsigargin (Tg), an activator of the integrated stress response. Chosen exposure time was before signal saturation in zebrafish samples.

CHOP (expected molecular weight - 29 kDa)

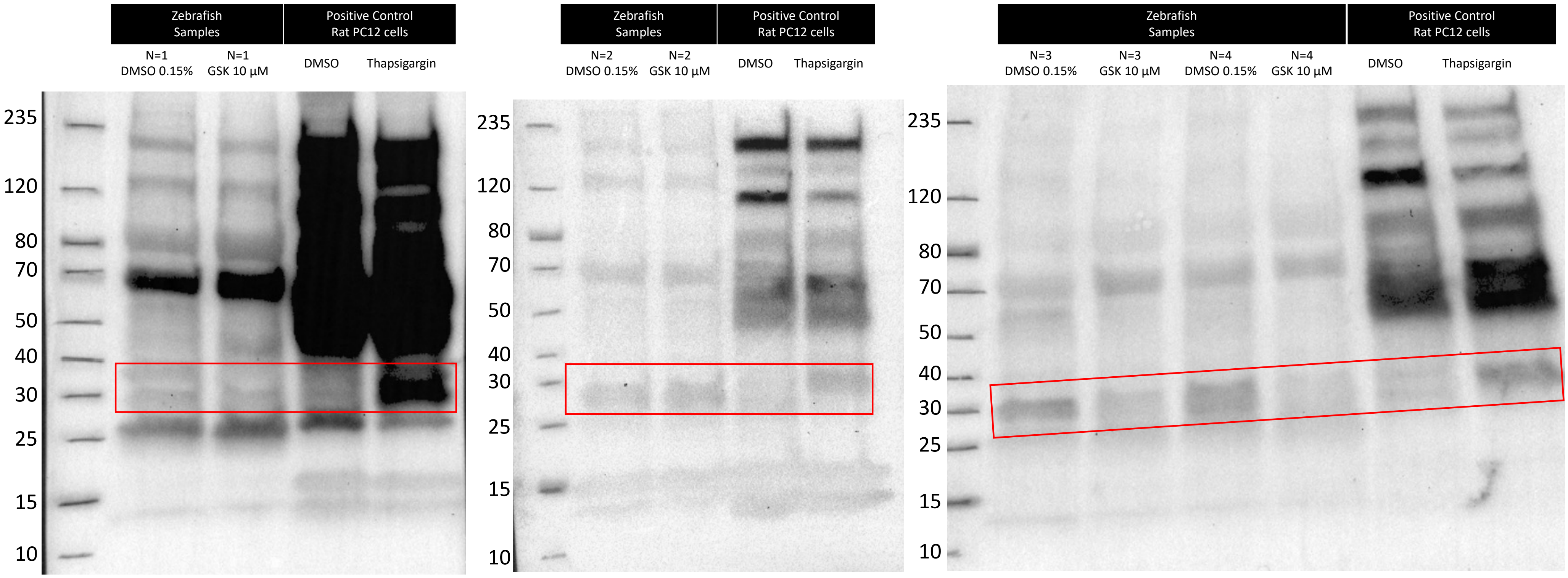

Supplementary Figure 7. Original western blots used for quantification of data in Fig. 2 D *vi*: red boxes show the quantified band. Band selection for zebrafish samples was made by comparison with positive control samples of PC12 cells incubated with thapsigargin (Tg), an activator of the integrated stress response. Chosen exposure time was before signal saturation in zebrafish samples.

Coomassie (total protein labelling)

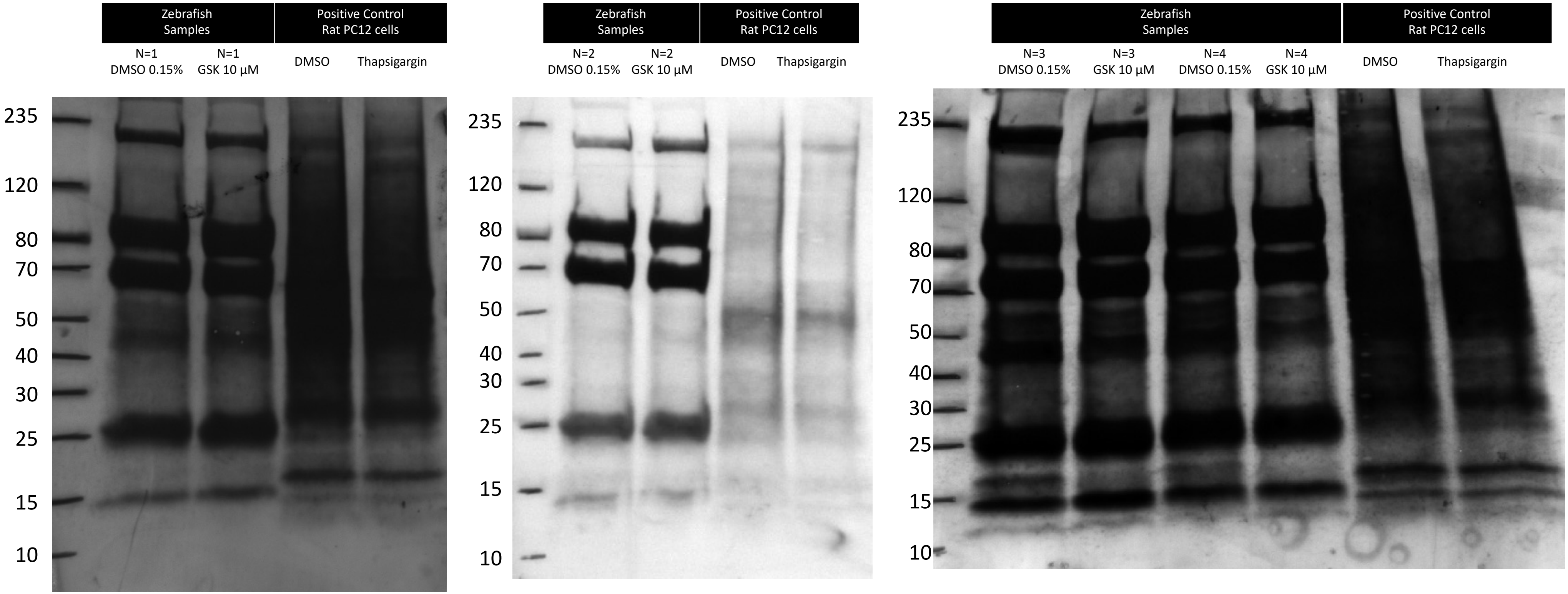

Supplementary Figure 8. Original western blot membranes stained with Coomassie used as loading control for quantification of data in Fig. 2 D *vi*.

Supplementary Table 1. Primary antibodies used in western blots from Fig. 2 D.

Antibody	Dilution	Supplier	Catalog Number	RRID
anti-ATF4	1:500	Proteintech	Cat# 10835-1-AP	AB_2058600
anti-CHOP	1:1000	Proteintech	Cat# 15204-1-AP	AB_2292610
anti-eIF2α	1:1000	Thermo Fisher Scientific	Cat# AHO0802	AB_2536316
anti-p-eIF2α	1:500	Thermo Fisher Scientific	Cat# MA5-15133	AB_10983400

Supplementary Table 2. Secondary antibodies used in western blots from Fig. 2 D.

Antibody	Dilution	Supplier	Catalog Number	RRID
anti-mouse	1:4000	Thermo Fisher Scientific	Cat# G-21040	AB_2536527
anti-rabbit	1:4000	Thermo Fisher Scientific	Cat# G-21234	AB_2536530
