# Supplementary figures and images for: The PERK inhibitor GSK2606414 evokes developmental defects in zebrafish consistent with Wolcott-Rallison syndrome phenotypes
Source: Pharmacol Rep. 2026 Feb 13;78(2):487–504. doi: 10.1007/s43440-026-00837-7 (PMC12975795; doi:10.1007/s43440-026-00837-7)

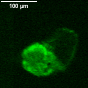

Supplement: Supplementary file 2 — Supplementary Material 2 [file 43440_2026_837_MOESM2_ESM.gif]

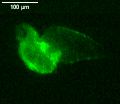

Supplement: Supplementary file 3 — Supplementary Material 3 [file 43440_2026_837_MOESM3_ESM.gif]
